# Effect of Nanoscale W Coating on Corrosion Behavior of Diamond/Aluminum Composites

**DOI:** 10.3390/nano13020307

**Published:** 2023-01-11

**Authors:** Ping Zhu, Qiang Zhang, Yixiao Xia, Kai Sun, Xiu Lin, Huasong Gou, Serge Shil’ko, Gaohui Wu

**Affiliations:** 1 School of Materials Science and Engineering, Harbin Institute of Technology, Harbin 150001, China; 2 Key Laboratory of Advanced Structure-Function Integrated Materials and Green Manufacturing Technology, Harbin Institute of Technology, Harbin 150001, China; 3 Industrial Technology Research Institute of Heilongjiang Province, Harbin 150001, China; 4 V.A. Belyi Metal-Polymer Research Institute of National Academy of Sciences of Belarus, 246050 Gomel, Belarus

**Keywords:** metal-matrix composites (MMCs), corrosion resistance, microstructures, interface reaction, metal coatings

## Abstract

The stability of diamond/aluminum composite is of significant importance for its extensive application. In this paper, the interface of diamond/aluminum composite was modified by adding nanoscale W coating on diamond surface. We evaluated the corrosion rate of nanoscale W-coated and uncoated diamond/aluminum composite by a full immersion test and polarization curve test and clarified the corrosion products and corrosion mechanism of the composite. The introduction of W nanoscale coating effectively reduces the corrosion rate of the diamond/aluminum composite. After corrosion, the bending strength and thermal conductivity of the nanoscale W-coated diamond/aluminum composite are considerably higher than those of the uncoated diamond/aluminum composite. The corrosion loss of the material is mainly related to the hydrolysis of the interface product Al_4_C_3_, accompanied by the corrosion of the matrix aluminum. Our work provides guidance for improving the life of electronic devices in corrosive environments.

## 1. Introduction

At present, the effective heat dissipation of microprocessors and power semiconductors has become one of the technical bottlenecks facing the development of the electronic information industry [1]. Electronic packaging materials play the role of mechanical support, sealing protection and heat dissipation. Developing electronic packaging materials with high thermal conductivity, low expansion and good mechanical properties has become a goal pursued by all countries. Diamond possesses outstanding physical properties, with a room temperature thermal conductivity of 2200 W/(m·K) and a thermal expansion coefficient of 0.8 × 10^−6^/K. Compared with 2D materials such as graphene, no anisotropy exists in the packaging materials prepared by diamond [2,3,4]. The diamond-reinforced aluminum matrix composites developed thus become the representative of a new generation of thermal management materials. In the fabrication process, the interface reaction between diamond and aluminum may occur to form Al_4_C_3_. A small amount of Al_4_C_3_ can enhance the interface bonding and improve the thermal conductivity of the composite [5]. However, Al_4_C_3_ is brittle and easy to deliquescence, which seriously endangers the performance reliability of composites.

Extensive research is devoted to modifying the interface of diamond/aluminum composites by optimizing the preparation process, metal matrix alloying and surface treatment of diamond particles [6,7,8,9,10,11,12,13]. Tan et al. predicted that W and WC are the most promising candidate materials for interfacial modification of nanolayers by using the acoustic mismatch model [14]. Compared with sol-gel method and diffusion method, the coating obtained by magnetron sputtering method is complete and uniform [15,16]. Low temperature sputtering prevents the metal from forming carbides, ensuring that the coating phase is controllable. The research on the influence of W coating thickness on the thermal conductivity of the diamond (100 μm)/aluminum composite shows that the composite with 45 nm W coating possesses the highest thermal conductivity of 622 W/(m·K) [17], and the properties only decreased by 2.8% after 30 days of moisture treatment. The researchers believed that the thickness of W coating greater than 45 nm is beneficial to the mechanical properties and reliability of the material, along with the sacrifice of some thermal conductivity [18,19]. Zhang et al. prepared a 420 nm W-coated diamond/aluminum composite by hot pressing sintering method. Although the diamond particle size was as high as 400 μm, the highest thermal conductivity of the composite was only 475 W/(m·K) [15]. It may be attributed to the high interfacial thermal resistance introduced by too thick of a coating.

Interfacial phase composition is also an important factor influencing the thermal conductivity and performance stability of composites. Yang et al. [20] reported that the interfacial phases of WC modified diamond/aluminum composites vary at different infiltration temperatures. Al_4_C_3_ was produced at the interface of the composites at 1073 K, and the properties decreased by 21.8% after immersion in deionized water for 48 h. In our previous work [21], we clarified the influence of 50 nm W and 50 nm WC coating on the interface structure of diamond/aluminum composites. The coating of WC can inhibit the formation of Al_4_C_3_, but the large size of Al_5_W and the presence of amorphous carbon at the interface result in lower thermal performance than W coating composites. While improving the thermal conductivity of diamond/aluminum composite is considered, the performance stability of the composite cannot be ignored. Few research on the stability of diamond/aluminum composites have been reported [22,23,24], regarding the effects of temperature shock and hydrothermal treatment on diamond/aluminum composites. As far as we know, there is no report on the corrosion behavior of diamond/aluminum composites.

According to the research on the corrosion performance of ceramic reinforced aluminum matrix composites [25,26,27], the pores at the interface of the composite are favorable pitting locations. For diamond/aluminum composites, the addition of diamond particles destroys the interface continuity in the matrix. In corrosive environmental media, the aluminum and diamond in diamond reinforced aluminum matrix composites may form corrosive galvanic cells, thus causing damage to electronic devices and functional failure [28]. The corrosion resistance and stability of ceramics reinforced aluminum matrix composites have great limitations on their practical value and application range. In this paper, the interface of diamond/aluminum composite was modified by nanoscale W coating on the surface of diamond, and the uncoated diamond/aluminum composite was also prepared for comparison. We quantitatively and qualitatively studied the corrosion rate of diamond/aluminum composite by full immersion test and polarization curve test, analyzed the corrosion products and expounded the corrosion mechanism of the composite, and verified the effectiveness of introducing nanoscale W coating to improve the mechanical properties and corrosion resistance of diamond/aluminum composite, and significantly improved the thermal conductivity stability of the composite in the corrosive environment.

## 2. Materials and Methods

### 2.1. Sample Preparation

**Figure 1:**
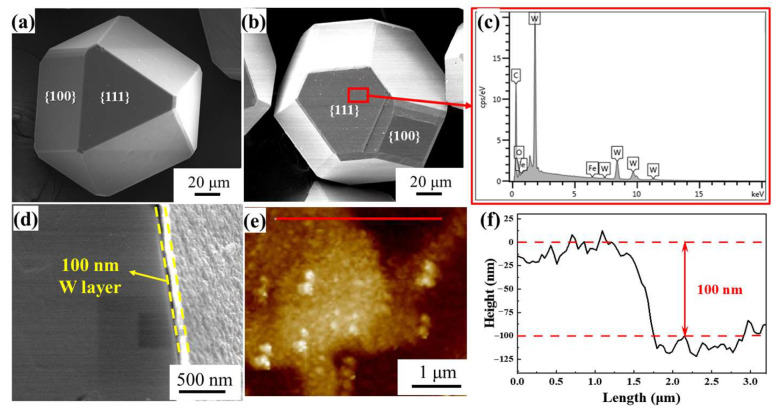
The morphology of diamond particles (**a**) uncoated diamond; (**b**) W-coated diamond; (**c**) EDS analysis results of the red areas in (**b**); (**d**) surface morphology of W-coated diamond particles after coating destruction; (**e**,**f**) diamond morphology characterized by AFM.

Raw materials used for composite were MBD4-grade synthetic monocrystalline diamond particles (~100 μm, Henan Huanghe Whirlwind Co., Henan, China) and commercial aluminum (99.99 wt.% in purity, Northeast Light Alloy Co., Ltd., Harbin, China). A 100 nm-thick tungsten coating was deposited onto the diamond particles by magnetron sputtering technique (MSP-5100B system, Beijing, China). A circular W target with the dimension of Φ 100 mm × 50 mm and purity of 99.99% was used for sputtering. During magnetron sputtering deposition, constant Ar flow was maintained to ensure that sputtering pressure was within the range of 5 × 10^−3^~9 × 10^−3^ Pa. The sputtering current and voltage were set at 0.9 A and 600 V, respectively, and 100 nm W-coated diamond particles were obtained by sputtering for 180 min at 400 °C. To ensure the uniformity of the coating, the sample holder swings 35 times per minute. The morphology of diamond particles before and after sputtering W coating is shown in Figure 1. Compared with other coating methods, the W coating deposited by magnetron sputtering method is uniform and complete. Figure 1d shows the thickness of 100 nm W coating by magnetron sputtering. It should be noted that the coating is artificially damaged to characterize the thickness of the coating. Atomic force microscopy (AFM) (Bruker Corporation, Karlsruhe, Germany) results further indicate that the thickness of the W coating is about 100 nm (Figure 1e).

W-coated diamond/aluminum composite with a 60% diamond volume fraction was fabricated by gas assisted pressure infiltration method, and uncoated diamond/aluminum composite was also fabricated by the same process. The infiltration parameters were set as 700 °C for 30 min, and the heating rates were all 30 °C/min, then pressurize until the pressure reached 15 MPa to complete the infiltration process. After that, the composites were furnace-cooled to room temperature and diamond/aluminum composites were obtained.

### 2.2. Corrosion Test

A 3.5 wt.% NaCl solution was selected as the corrosion medium according to the marine atmosphere. The sample used for corrosion test were cylinder-shaped specimen with 12.7 mm in diameter and 3 mm in thickness, and three patterns of each composite were selected for testing. The solution was changed every seven days. After taking out the sample, rinse with distilled water to observe the corrosion morphology of the sample surface. The corrosion products were then wiped off and ultrasonic cleaned in distilled water. After drying, the composite was accurately weighed by a balance with an accuracy of 0.1‰, and the corrosion rate of the composite can be calculated by Equation (1):

(1) $$R=\frac{8.76\times 10^{7}\times \left(M-M_{1}\right)}{\mathrm{STD}}$$

where R is the corrosion rate, M and M_1_ are the sample mass before and after corrosion, S is the total area of the sample, T is the test time, and D is the density of the sample.

The total corrosion weight loss time was determined according to the corrosion rate. Therefore, the corrosion rate was calculated by pre-etching the uncoated and W-coated diamond/aluminum composites for 24 h to determine the final corrosion time and set the corrosion intermittent time point. The polarization curves of uncoated and W-coated diamond/aluminum composites were measured by using PARCM332 system (Shanghai, China).

### 2.3. Characterization

X-ray diffraction (Rigaku Corporation, Tokyo, Japan) was used to investigate the phase composition of the diamond/Al composites before and after corrosion at a scan rate of 10 °/min, using Cu-Kα radiation. Microstructural characterization of composites was performed by using a field-emission scanning electron microscope (HELIOS NanoscaleLab 600i, Hillsboro, OR, USA). Three-point bending test was completed by Instron 5569 (Instron, Boston, MA, USA) universal electrical tensile testing machine, with a loading rate of 0.5 mm/min and a span of 30 mm. The dimensions of the samples were 3 mm × 4 mm × 36 mm. The thermal diffusion coefficient k of diamond/aluminum composite was measured by Netzsch LFA 467 Nanoflash instrument (Netzsch, GmbH, Selb, Germany) with the sample dimension of Φ12.7 mm × 3 mm. The thermal conductivity of the composites was determined by the formula λ = k·ρ·c, where ρ and c represent the density and specific heat capacity of the composite, respectively.

## 3. Results and Discussion

### 3.1. Interface Phase and Morphology of Nanoscale Coated Diamond/Aluminum Composites

As mentioned above, the diamond/aluminum composites are easy to form into the interface product Al_4_C_3_. Although it is beneficial to interface bonding, it greatly harms the performance stability of composites. Coating on diamond surface is an effective method to improve the selectivity of diamond/aluminum interface and inhibit the formation of Al_4_C_3_. Among Ti, Cr, Mo, W several carbide-forming elements, element W, which has the highest thermal conductivity, is a favorable candidate for coating elements. Yang et al. [17] reported that the diamond/aluminum composite modified by the 45 nm W coating has the best thermal conductivity. To obtain better mechanical properties and corrosion resistance, we choose to increase the thickness of W coating. However, a too thick coating brings additional interface thermal resistance, which will lead to a sharp decrease in the thermal conductivity of the composite. It is necessary to modify the interface of diamond/aluminum composite with nanoscale W coating, so the coating thickness is set as 100 nm.

**Figure 2:**
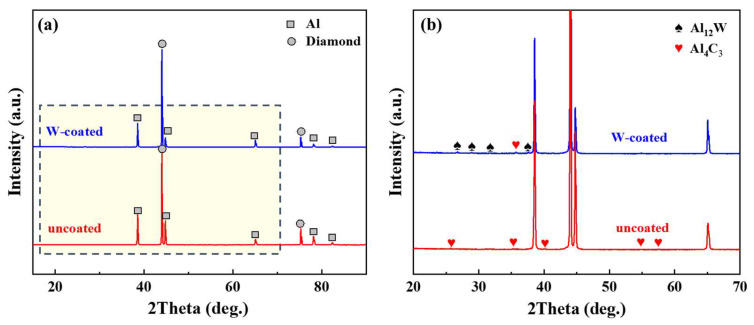
XRD pattern of diamond/aluminum composite: (**a**) Total; (**b**) Local.

Figure 2 shows the XRD patterns of diamond/aluminum composites without and with W coatings. The diffraction peaks of diamond and aluminum were detected in the composites. It can be observed that Al_4_C_3_ is detected in both uncoated and W-coated diamond/aluminum composites by local amplification of the spectrum. The number and intensity of Al_4_C_3_ diffraction peaks in W-coated diamond/aluminum composite are significantly reduced, suggesting that the content of Al_4_C_3_ in W-coated diamond/aluminum composite is less than that in uncoated diamond/aluminum composite. In addition, the interface product Al_12_W was also detected in the W-coated diamond/aluminum composite.

The possible intermetallic compounds formed by the reaction between aluminum and tungsten are Al_4_W, Al_5_W and Al_12_W [29]. For W-coated diamond/aluminum composites, the possible interface phases are generally Al_5_W and Al_12_W [30]. It is reported that when the infiltration temperature is 750 °C, Al_12_W completely disappears and Al_5_W is formed [20]. The preparation temperature in this paper is 700 °C, so the interface phase formed is more inclined to be Al_12_W. In addition, the temperature is not high enough for the reaction between diamond and tungsten. In the preparation process of composites, aluminum and tungsten react preferentially to form Al_12_W, which reduces the contact time between diamond and aluminum. However, the consumption of W coating makes the diamond and aluminum inevitably form a small amount of Al_4_C_3_.

**Figure 3:**
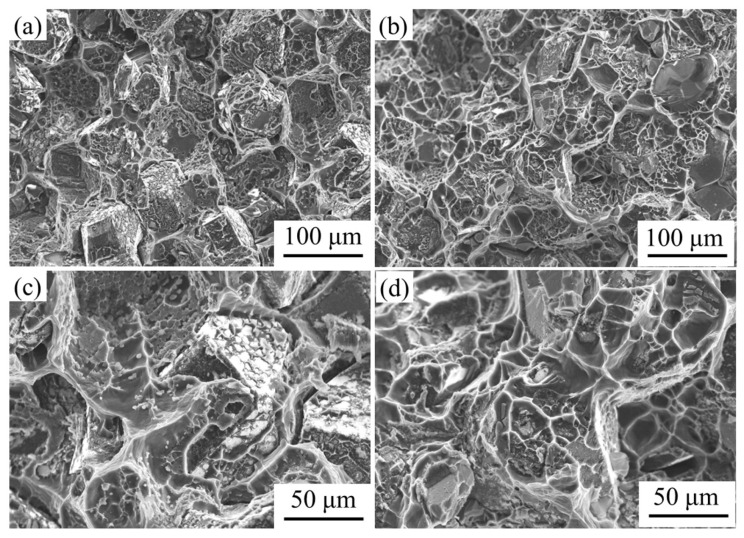
Fracture morphology of diamond/aluminum composites: (**a**,**c**) uncoated diamond/aluminum composites; (**b**,**d**) W coated diamond/aluminum composite.

Figure 3 shows the fracture morphology of diamond/aluminum composites. According to Figure 3a, in uncoated diamond/aluminum composites, the bonding between different crystal surfaces of diamond and aluminum matrix presents obvious interfacial selectivity. Due to the short heat preservation time, there is no chemical reaction between diamond {111} and aluminum, which shows mechanical bonding, while diamond {100} can react with aluminum to generate blocky Al_4_C_3_. Al_4_C_3_ generated in situ can pin the interface, resulting in strong interface binding.

However, the fracture surface of W-coated diamond/aluminum composite presents microporous aggregation fracture, and a large number of dimples can be observed, in which some cracked second phases are distributed. Combined with the above XRD analysis, it was determined that the substance was Al_12_W. The fracture occurs in the aluminum matrix, indicating that the introduction of nanoscale-W coating improves the interface bonding between diamond and aluminum. At the same time, both {100} and {111} crystal faces of diamond adhered to aluminum, which improved the selective binding of diamond to aluminum.

### 3.2. Corrosion Behavior of Diamond/Aluminum Composites

The corrosion rates of uncoated and W-coated diamond/aluminum composites immersed in 3.5 wt.% NaCl solution for 24 h were 1.21 mm/a and 0.09 mm/a, respectively. The corrosion rate of uncoated diamond/aluminum is higher. In order to better detect the change of corrosion weight loss with time, the sampling time should be shortened in the early stage. The corrosion tendency of W-coated diamond/aluminum composite is small, so the sampling time can be appropriately extended. The corrosion time is determined according to the corrosion rate. Therefore, the corrosion time of the diamond/aluminum composite without coating and W coating is 144 h and 264 h, respectively. In addition, to ensure that the corrosion environment is almost unchanged at each corrosion stage, the solution should be replaced after 7 days of corrosion if the corrosion time exceeds 7 days.

**Figure 4:**
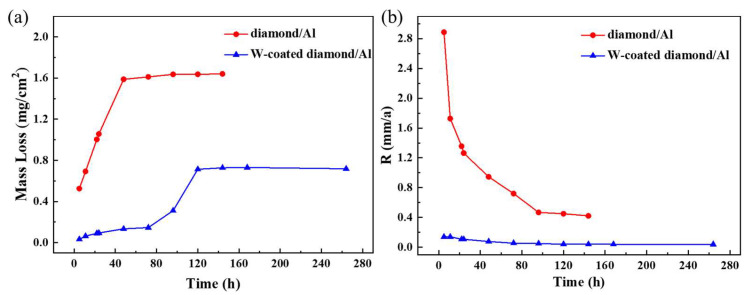
(**a**) Corrosion weight loss-time curve and (**b**) Corrosion rate-time curve of diamond/aluminum composite.

The change curves of corrosion weight loss and corrosion rate are shown in Figure 4a,b, respectively. The corrosion rate of the uncoated diamond/aluminum composite is significantly higher than that of the W nanoscale coated diamond/aluminum composite. For uncoated diamond/aluminum composites, the mass loss of the composite increases almost linearly when the soaking time is 0~48 h. Further extension of soaking time, the mass loss is only slightly changed. The calculation of corrosion rate also suggests that the corrosion rate stabilizes around 0.4 mm/a after soaking time exceeds 96 h. The corrosion rate of the composite is dominated by the interface product Al_4_C_3_. The uncoated diamond/aluminum composite forms a large amount of Al_4_C_3_ at the diamond/aluminum interface, which is easily hydrolyzed to CH_4_ in the corrosive medium, leaving gaps at the interface and causing large mass loss. The hydrolysis of Al_4_C_3_ results in the preferential pitting and nucleation of the composite at the interface, on which the pitting expands and corrodes along the interface, making interface denudation.

After the diamond surface is coated with W, the linear relationship of the mass loss of the composite is different when the immersion time is 0~72 h and 72~120 h. In the early stage, the mass loss is mainly caused by the hydrolysis of a small amount of Al_4_C_3_ in the composite, while in the later stage, it is mainly caused by the corrosion of Al matrix. It is worth noting that the NaCl solution was replaced on the seventh day of the full immersion test of the W-coated composite. However, the mass loss did not further increase, and the calculated corrosion rate also approached zero. Therefore, the introduction of nanoscale W coating effectively reduces the corrosion rate of diamond/aluminum composite.

**Figure 5:**
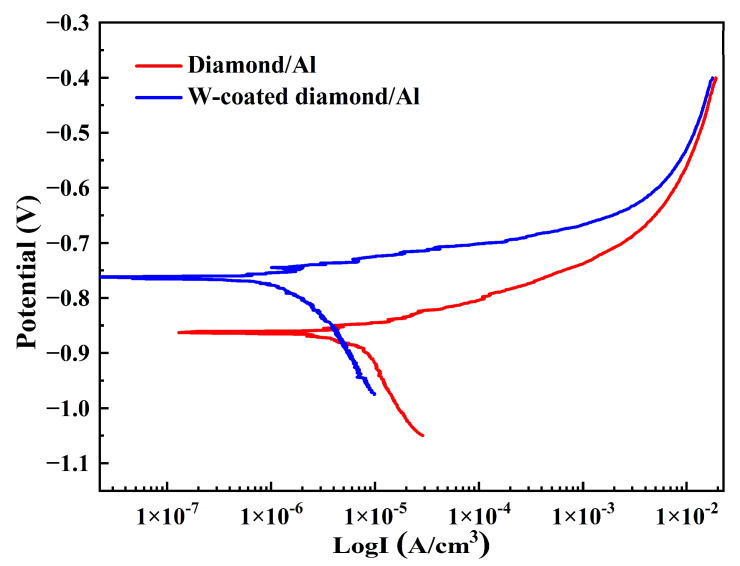
Polarization curve of diamond/aluminum composite.

In order to further study the corrosion behavior of diamond/aluminum composites, the polarization curves of diamond/aluminum composites were measured, and the results were shown in Figure 5. The corrosion potential (E_corr_) of W-coated diamond/aluminum composite is greater than that of uncoated diamond/aluminum composite, so W-coated diamond/aluminum composite is less likely to corrode. From the perspective of corrosion current density (I_corr_), the uncoated diamond/aluminum composite has a larger I_corr_, so the corrosion rate is faster. It is consistent with the result of corrosion weightlessness. The corrosion test shows that although the nanoscale-coating reacts with aluminum matrix to form Al_12_W in the process of composite preparation, the introduction of coating reduces the corrosion tendency of diamond/aluminum composite and improves the corrosion resistance of the composite.

**Figure 6:**
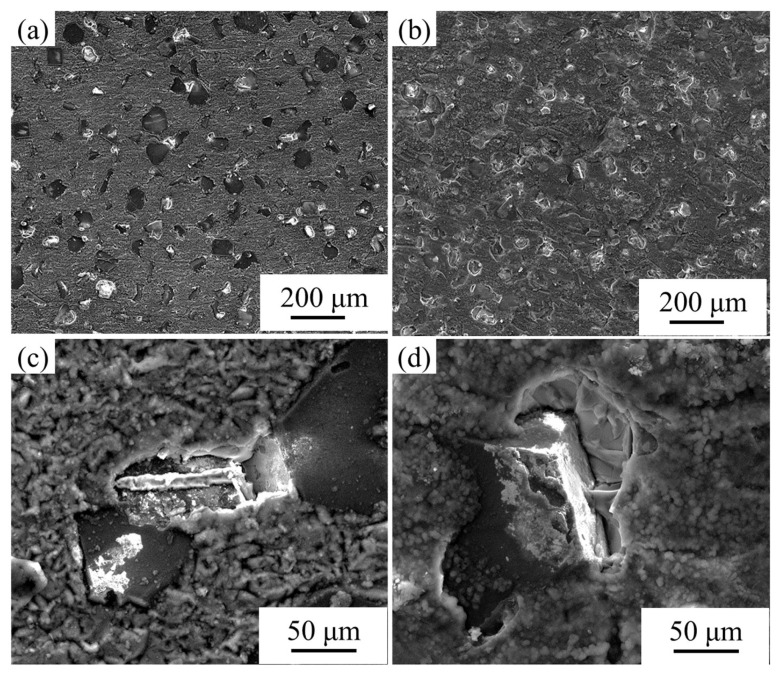
Surface morphology of diamond/aluminum composite after corrosion: (**a**,**c**) uncoated diamond/aluminum composite; (**b**,**d**) W-coated diamond/aluminum composite.

Figure 6 shows the surface morphology of the composites after corrosion. For the uncoated diamond/aluminum composite, the etch pits are mostly distributed at the interface between diamond and aluminum, that is, the interface is the site where pitting preferentially nucleates. Al_4_C_3_, with hygroscopic characteristics, will react with water to form Al(OH)_3_. No interface product Al_4_C_3_ is found in the etch pit. The site of Al_4_C_3_ hydrolysis was the starting point of pit nucleation, and the pit gradually increased and expanded with the corrosion. For W-coated diamond/aluminum composite, there are also some corrosion pits at the edges of diamond, which is caused by a small amount of Al_4_C_3_ at the interface. However, the corrosion at the interface is weaker than that of the uncoated diamond/aluminum composite, and the aluminum matrix is also partially corroded, exposing diamond particles buried below.

**Figure 7:**
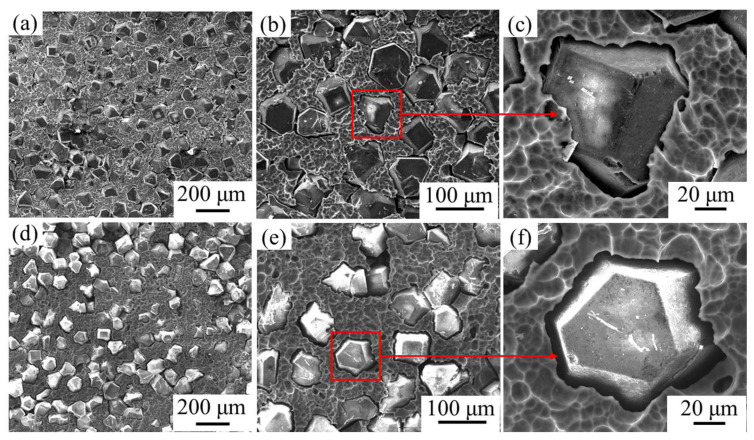
Surface morphology of diamond/aluminum composite after electrified accelerated corrosion: (**a**–**c**) uncoated diamond/aluminum composite; (**d**–**f**) W-coated diamond/aluminum composite.

The surface morphology of diamond/aluminum composite after electric accelerated corrosion is shown in Figure 7. For uncoated diamond/aluminum composite, most diamond particles are wrapped by aluminum matrix before corrosion. After corrosion, aluminum on the surface is dissolved and a large number of diamond particles are exposed. The corrosion mainly occurs in the aluminum matrix and the interface between diamond and aluminum, and the corrosion form is interface denudation. Electrification accelerates the hydrolysis of Al_4_C_3_, and an obvious gap appears between the diamond and aluminum.

The surface morphology and corrosion form of W-coated diamond/aluminum composite after corrosion are similar to those of uncoated diamond/aluminum composite, which is attributed to the existence of some interface products Al_4_C_3_ at the interface of the composite. After corrosion, the diamond surface is clean and there is no residual interface product, indicating that Al_4_C_3_ reacts preferentially with the corrosive medium in the corrosion stage. It is speculated that some intermetallic compounds exist in the aluminum matrix and peel off with the corrosion of the surface aluminum. In addition, after the corrosion of the interface products in the W-coated diamond/aluminum composite, the corrosion mainly occurs in the aluminum matrix, so there is a large area of exposure of diamond particles.

### 3.3. Analysis of Corrosion Products

**Figure 8:**
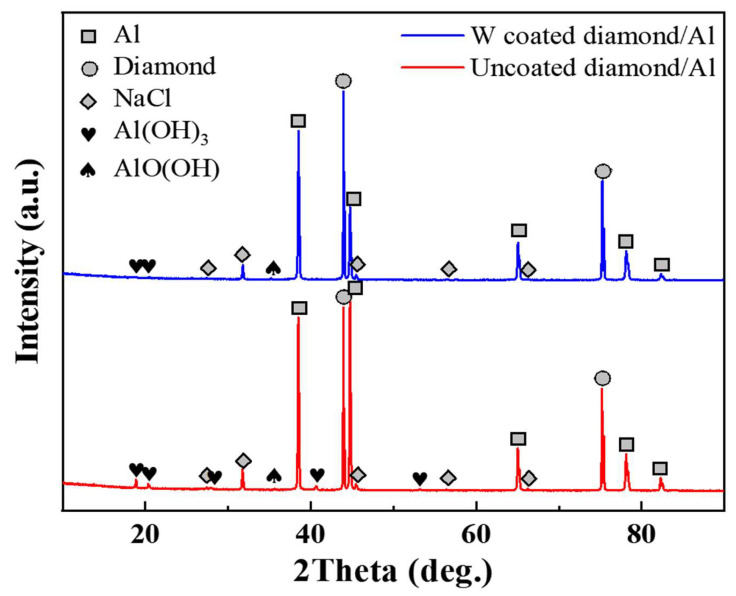
XRD pattern of diamond/aluminum composite after corrosion.

**Figure 9:**
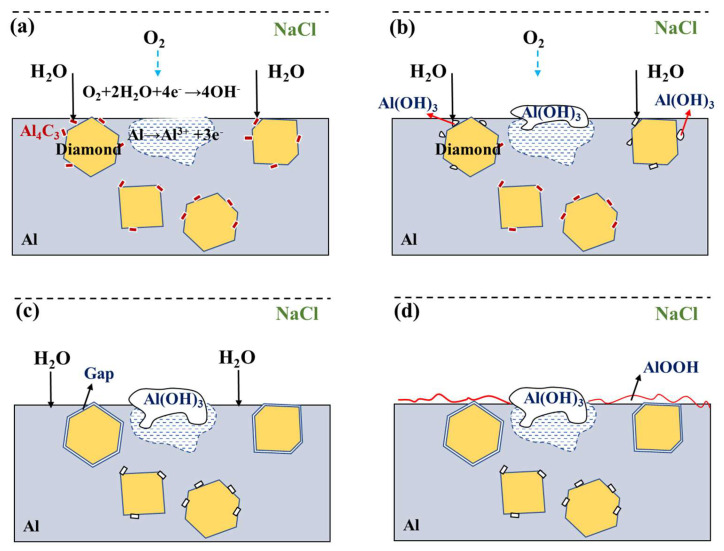
Schematic diagram of corrosion process of diamond/aluminum composite: (**a**) hydrolysis of Al_4_C_3_ and corrosion of aluminum; (**b**) formation and accumulation of corrosion product Al(OH)_3_; (**c**) corrosion creates gaps between diamond and aluminum; (**d**) the oxidation reaction of aluminum matrix forms AlOOH to inhibit further corrosion.

Figure 8 shows the XRD pattern of the corroded diamond/aluminum composite. In addition to the diffraction peaks of diamond and Al, we also detected NaCl, Al(OH)_3_ and AlOOH in the composite. The corrosion medium NaCl was detected because the product on the sample surface was not cleaned during the test of XRD. According to the intensity and amount of diffraction peak, the corrosion product Al(OH)_3_ in the uncoated diamond/aluminum composite is more than that in W-coated diamond/aluminum composite. Figure 9 depicts the corrosion process of diamond/aluminum composite.

First, the composite was immersed in NaCl solution. Due to the electrode potential difference between diamond and aluminum, Al_4_C_3_, which is easy to hydrolyze, is generated. Corrosion preferentially occurs at the interface of diamond/aluminum composites, where Al_4_C_3_ reacts with water to form Al(OH)_3_. The content of Al_4_C_3_ in W-coated diamond/aluminum composite is less, so the corrosion product Al(OH)_3_ is also less than that of uncoated diamond/aluminum composite, corresponding to the weakening of the intensity of Al_4_C_3_ diffraction peak in XRD and the reduction of the number of diffraction peaks.

Al_4_C_3_ + H_2_O → Al(OH)_3_↓ + CH_4_↑ (2)

In addition, electrochemical corrosion of Al also occurs in NaCl solution, and the reaction is as follows:

Al → Al^3+^ + 3e^−^ (3)

O_2_ + 2H_2_O + 4e^−^ → 4OH^−^ (4)

The total reactions occurred are as follows:

4Al + 3O_2_ + 6H_2_O → 12Al(OH)_3_ (5)

Therefore, the corrosion reaction occurs at the interface and on the surface of the aluminum matrix, and the product is Al(OH)_3_, as shown in Figure 9b. Due to the hydrolysis reaction of Al_4_C_3_ between diamond and aluminum, the nearby Al matrix is also corroded as the corrosion continues. Therefore, with the extension of immersion time, there is a gap between diamond and aluminum, and the corrosion form is interface denudation. The corrosion product Al(OH)_3_ covers the surface of the composites, which hinders the exchange of this area with other substances and inhibits the electrochemical corrosion, as shown in Figure 9c.

On the other hand, as described in Figure 9d, with the progress of corrosion, aluminum is oxidized in the solution, and a layer of Al_2_O_3_·H_2_O(AlOOH) is formed on its surface, which further passivates aluminum. Therefore, after the corrosion reaches a certain time, the corrosion weight loss is almost unchanged. The electrochemical reaction of this process is as follows:

Al+ H_2_O → AlOH + H+ + e^−^ (6)

AlOH + H_2_O → Al(OH)_2_ + H^+^ + e^−^ (7)

Al(OH)_2_ →AlO(OH) + H^+^ + e^−^ (8)

According to the above analysis, the corrosion process of the uncoated diamond/aluminum composite mainly includes the hydrolysis of Al_4_C_3_ and the oxygen absorption corrosion of aluminum. As the uncoated diamond/aluminum composite contains more Al_4_C_3_, the hydrolysis of Al_4_C_3_ makes the interface between diamond and aluminum produce gaps, which promotes the further corrosion. A similar situation has been reported in aluminum matrix composites reinforced with graphite or silicon carbide. Al_4_C_3_ generated by the reaction at the interface will be hydrolyzed in the electrolyte to accelerate the corrosion rate of the composite [31,32]. 

During the preparation of W-coated diamond/aluminum composite, part of W coatings diffuses into the Al matrix to form intermetallic compounds, there are still some W coatings on the diamond surface. The introduction of W coatings reduces the contact time between the diamond and aluminum, and inhibits the generation of Al_4_C_3_. In the corrosion process of the W-coated diamond/aluminum composite, aluminum galvanic corrosion mainly occurs due to the low content of Al_4_C_3_ and good corrosion resistance of W. Therefore, the diffraction peak intensity of Al(OH)_3_ in W-coated diamond/aluminum composite after corrosion is significantly lower than that of uncoated diamond/aluminum composite. With the extension of corrosion time, the corrosion product Al(OH)_3_ on the surface of the composite and the product AlOOH of the oxidation of the aluminum matrix inhibit the continuation of corrosion. The mass loss caused by corrosion almost remains unchanged, and the corrosion rate decreases until it tends to zero.

### 3.4. Effect of Corrosion on Bending Strength and Thermal Conductivity of Diamond/Aluminum Composite

Compared with aluminum alloy, the reinforcement will destroy the passive film on the surface of the matrix alloy, increasing the location of corrosion initiation and cracks [27,33]. The corrosion test of aluminum matrix composites mainly includes potentiodynamic polarization measurement and immersion test. Regarding corrosion resistance of aluminum matrix composites, recent studies are mainly focused on the influence of different alloy content, reinforcement volume fraction and other factors on the corrosion rate of the composites, with less attention on mechanical and functional characteristics. In addition to the full immersion test and polarization curve test to study the corrosion performance of the diamond/aluminum composite, we also further analyzed the influence of corrosion on the bending strength and thermal conductivity of the diamond/aluminum composite.

**Figure 10:**
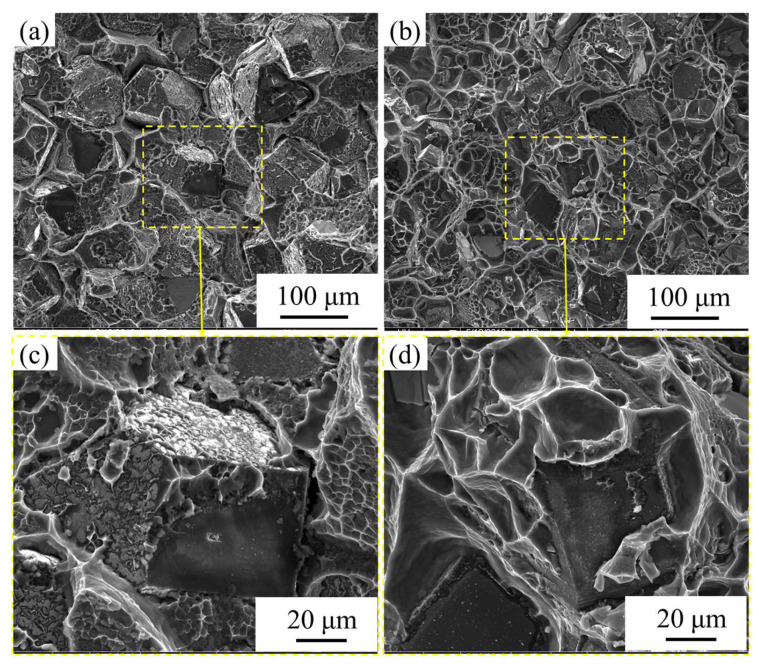
Fracture morphology of diamond/aluminum composite after corrosion: (**a**,**c**) uncoated; (**b**,**d**) nanoscale W-coated.

The mechanical properties of diamond/aluminum composites are greatly attenuated due to corrosion. After full immersion corrosion treatment, the bending strength of the uncoated diamond/aluminum composite decreased from 222 MPa to 180 MPa, and the bending strength of the W-coated diamond/aluminum composite decreased from 319 MPa to 195 MPa. Compared with diamond/aluminum composite (188 MPa), Cr-coated diamond/aluminum composite (137 MPa), W-coated diamond/aluminum composite (304 MPa), Ti coated diamond/aluminum composite (313 MPa) reported in the literature [6,18,34,35], the obtained W-coated diamond/aluminum composites showed a higher bending strength. Before and after corrosion, the bending strength of W-coated diamond/aluminum composite is higher than that of the uncoated diamond/aluminum composite. After corrosion, the bending strength of W-coated diamond/aluminum composite decreases greatly, up to 38.9%. Therefore, the fracture morphology of the composite after corrosion was further characterized, as shown in Figure 10.

The content of Al_4_C_3_ in the fracture surface of uncoated diamond/aluminum composite is obviously reduced, and the selective combination of different crystal planes of diamond with aluminum is reduced. Al_4_C_3_ on the diamond (100) crystal plane is hydrolyzed in the corrosive medium, which leads to the poor combination of diamond and aluminum, thus reducing the bending strength. Almost no Al_4_C_3_ was found at the fracture of the W-coated diamond/aluminum composite. Most of the diamond particles adhered to aluminum at the fracture, and the interface bonding was better than that of the uncoated diamond/aluminum composite with completely bare diamond. The addition of coating between metal and ceramic is conducive to good interface bonding, which enables the smooth transfer of heat flux or stress from the matrix to the reinforcement phase and obtains excellent mechanical and functional properties of the composite [36]. Nosewicz et al. reported that chromium, tungsten and molybdenum as coatings could improve the structure and performance of the metal/ceramic interface based on experiments and calculations [37].

It is worth noting that compared with the fracture morphology before corrosion, there are also some bare diamond particles in the composites with nanoscale W coatings after corrosion. It is attributed to the presence of a small amount of Al_4_C_3_ in the composites, which mainly grows on the {100} crystal of diamond. Therefore, some {100} crystal of diamond appears naked. On the other hand, because the introduction of W coating improves the selective combination of diamond and aluminum, the aluminum matrix is mainly corroded in the corrosive medium. The interfacial bond between different crystal surfaces of diamond and aluminum was destroyed. The layer on the surface of the composite, due to the corrosion of Al matrix near the interface and the shedding of intermetallic compounds with pinning effect, prefabricated crack initiation and nucleation sites for the composite, resulting in a decline in bending strength. 

The corrosion also led to the decrease of thermal conductivity of diamond/aluminum composites. The initial thermal conductivity of uncoated and 100 nm W-coated diamond/aluminum composites is 604 W/(m·K) and 579 W/(m·K), respectively. After full immersion corrosion, their thermal conductivity decreases by 12.7% and 4.2%, respectively. The thermal conductivity of 100 nm W-coated diamond/aluminum composites is slightly lower than that of uncoated ones, possible reasons are as follows. First, in order to prevent the too thin nano layer from being insufficient to improve the corrosion resistance of the composite, we selected the thickness of W coating as 100 nm. Compared with the thinner nano coating, additional interface thermal resistance is introduced. Secondly, part of the coating reacts with aluminum to form Al_12_W, which weakens the bridging effect of the coating. Finally, the introduction of the W coating inhibits the formation of Al_4_C_3_. According to the report of Yang et al. [20], for the W-coated diamond/aluminum composite, the interfacial phase prepared at a higher impregnation temperature (1073 K) contains Al_4_C_3_, and its thermal conductivity is higher than that of the composite prepared at a lower impregnation temperature (1023 K) with interfacial phases of Al_5_W and WC. 

After full immersion, due to the hydrolysis of Al_4_C_3_, the interfacial integrity of the uncoated diamond/aluminum composite is seriously damaged, resulting in a sharp decline in thermal performance. It can be seen from the fracture after corrosion that, the internal interface integrity of W-coated diamond/aluminum composite is relatively intact, with less performance attenuation. Li et al. reported that the thermal conductivity of diamond/aluminum composites without coating and SiC coating decreased by 22.5% and 6.3%, respectively, after soaking in deionized water for 500 h [38]. In the fracture of uncoated diamond/aluminum composite prepared by Li et al., it can be found that {100} diamond surfaces are almost completely covered by Al_4_C_3_, indicating that there is a large amount of Al_4_C_3_, which leads to such a serious decline in performance. In this work, by modifying diamond particles with nanoscale W coating, the decline of thermal conductivity of composites in a corrosive environment has been effectively restrained, ensuring the performance stability of materials in a corrosive environment. 

## 4. Conclusions

In this paper, the corrosion resistance of diamond/aluminum composite was improved by coating nanoscale W layer on the diamond surface. Diamond/aluminum composites without coating and nanoscale W coating were prepared by gas assisted pressure infiltration method. The introduction of nanoscale W coating reduces the contact time between diamond and aluminum matrix, effectively improves the interface bonding between diamond and aluminum, and inhibits the formation of interface product Al_4_C_3_. The full immersion experiment showed that the corrosion weight loss of W nano coating diamond/aluminum composite was lower than that of uncoated diamond/aluminum composite. The polarization curve measurement also verified the effectiveness of nanoscale W coating in reducing the corrosion rate of the composite. The corrosion of diamond/aluminum composites is mainly caused by the hydrolysis of Al_4_C_3_ and the corrosion of Al matrix in the corrosion medium. The aluminum matrix also has an oxidation reaction in the corrosion medium, forming AlOOH. The bending strength of W-coated diamond/aluminum composite can reach 319 MPa, which is still higher than that of uncoated diamond/aluminum composite after corrosion. Full immersion corrosion reduces the thermal conductivity of uncoated and W-coated diamond/aluminum composites by 12.7% and 4.2%, respectively. The addition of nanoscale W coating can improve the corrosion resistance of diamond/aluminum composite and contribute more to improving the thermal conductivity stability in corrosive environment. 

## Data Availability

Data presented in this article are available at request from the corresponding author.
